# Flow-induced choking of a compliant Hele-Shaw cell

**DOI:** 10.1073/pnas.2008273117

**Published:** 2020-11-16

**Authors:** Finn Box, Gunnar G. Peng, Draga Pihler-Puzović, Anne Juel

**Affiliations:** ^a^Department of Physics & Astronomy, University of Manchester, Manchester M13 9PL, United Kingdom;; ^b^Manchester Centre for Nonlinear Dynamics, University of Manchester, Manchester M13 9PL, United Kingdom;; ^c^Department of Applied Mathematics and Theoretical Physics, University of Cambridge, Cambridge CB3 0WA, United Kingdom

**Keywords:** choking, bulging, flow, deformation, fuse

## Abstract

After centuries of striving for structural rigidity, engineers and scientists alike are increasingly looking to harness the deformation, buckling, and failure of soft materials for functionality. In fluidic devices, soft deformable components that respond to the flow have the advantage of being passive; they do not require external actuation. Harnessing flow-induced deformation for passive functionality provides a means of developing flow analogs of electronic circuit components such as fluidic diodes and capacitors. The electronic component that has so far been overlooked in the microfluidics literature—the fuse—is a passive safety device that relies on a controlled failure mechanism (melting) to protect a circuit from overcurrent. Here, we describe how a compliant Hele-Shaw cell behaves in a manner analogous to the electrical fuse; above a critical flux, the flow-induced deformation of the cell blocks the outflow, interrupting (choking) the flow. In particular, the pressure distribution within the fluid applies a spatially variant normal force to the soft boundary, which causes nonuniform deformation. As a consequence of lateral confinement and incompressibility of the soft material, this flow-induced elastic deformation manifests as bulging near the cell outflow; bulges that come into contact with the rigid cell roof interrupt the flow. We identify two nondimensional parameters that govern the central deflection and the choking of the cell, respectively. This study therefore provides the mechanical foundations for engineering passive-flow limiters into fluidic devices.

## Significance

Harnessing flow–structure interactions has enabled the development of fluidic analogs of electronic circuit components, e.g., fluidic capacitors that store fluid much like an electrical capacitor stores charge. These soft components function in response to the flow; integrated into microfluidic devices, they remove the need for external actuation, thereby facilitating deployment outside of the laboratory. We describe a fluidic fuse that exploits the flow-induced deformation of a soft elastomer and identify the critical flow rate above which the flow is interrupted. This paves the way for the integration of passive flow limiters into microfluidic devices.

Microfluidic devices have far-ranging applications in industries as diverse as agriculture (1), tissue engineering (2), fertility treatment (3), food safety (4, 5), and cancer research (6, 7). A fundamental challenge in the deployment of microfluidic devices outside of the laboratory lies in removing the dependence of these lab-on-chip devices on off-chip hardware, such as intricate plumbing systems and external actuators. Progress has been made through integration of deformable features that serve as analogs of electronic circuits (8). For example, a bed of soft hairs tethered to channel walls at an angle provides low resistance to flow in one direction and high resistance to flow in the opposite direction, in a manner analogous to an electronic diode (9), because flow with the grain bends hair tips toward the channel walls, whereas flow against the grain bends hair tips toward the channel center. Other examples include fluidic valves and pumps (10–12), diodes and capacitors (13), resistors (circuits with tunable flow resistance) (14), and transistors (15, 16). Pertinent to this study is the fluidic analog of a current-dependent switch (17), which consists of two channels in parallel. In one, a portion of wall is replaced by an elastic arch that curves inwards and acts as a constriction, so fluid flows through the other, rigid channel. Above a critical flux, flow-induced “snap-through” of the elastic arch—to an unconstricting configuration—can divert flow from the rigid channel to the flexible channel.

As in ref. 17, functional integration of deformable components has typically relied on thin membranes, or slender bodies. We instead consider a thick, elastic boundary within a rigid enclosure and examine how flow-induced deformation of the confined elastomer generates localized regions of flow constriction. We draw parallels with the electrical fuse—a ubiquitous component that interrupts the flow of current when a threshold value is exceeded and thereby allows safe working levels to be engineered into electrical circuits. The microfluidic equivalents of current and voltage are flow rate and fluid pressure; here, we show that flow rates that exceed a threshold value can induce channel closure and reduce the outflux to zero (interrupting the fluidic circuit). The failure mechanisms differ in the two devices, however, since elastic deformation causes choking of the flow in our soft cell, whereas Joule heating causes the wire in a fuse to melt.

**Fig. 1.**
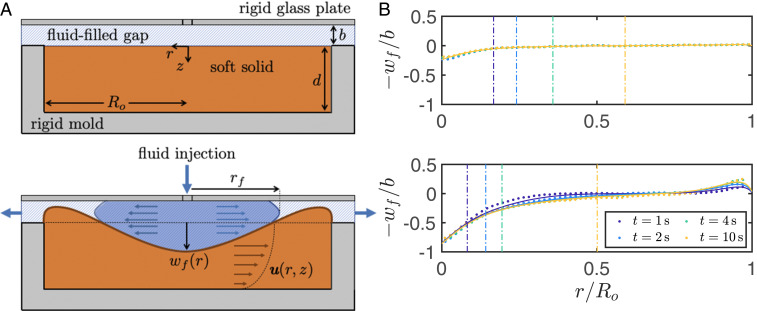
Designing a fluidic fuse. (*A*) Schematic diagrams of a compliant Hele-Shaw cell: a fluid-filled, narrow gap between a rigid, horizontal plate and a slab of soft solid that is confined within a rigid mold (*Top*). Dyed fluid injected into the compliant Hele-Shaw cell at a constant flow rate spreads outwards, axisymmetrically with a radial front position $r_{f}\left(t\right)$, deforming the soft solid and displacing fluid resident in the cell (*Bottom*). For sufficiently high fluid–structure interaction parameter, the bulges near the cell rim make contact with the cell roof, blocking the outflow and interrupting the flow in a manner analogous to an electrical fuse. (*B*) Deflection profiles of the free surface of the solid scaled by the initial gap thickness, $-w_{f}/b$, as a function of radial position normalized by the cell radius, $r/R_{o}$, for slab aspect ratio $A=R_{o}/d=8.57$ and FSI parameter $F=12\mu Qd/\left(2\pi Gb^{4}\right)=0.79$ (*Top*) and F=6.64 (*Bottom*). The different color profiles show the evolution of the deformation profile in time (as indicated in the legend). Experimental and numerical data (available in Dataset S1) are shown by markers and solid lines, respectively; vertical dashed lines indicate the radial position of the front of intruding fluid $r_{f}$ at the corresponding instants in time in the experiments. Numerical simulations conserve volume exactly, while the measured solid volume in the experiments is constant within experimental tolerances.

The fluidic system under study is a soft Hele-Shaw cell—a fluid-filled, narrow gap between a rigid plate and an elastic solid that is confined inside a rigid mold, as shown in Fig. 1*A*. Soft slabs of radius $R_{o}=60$ mm and depths d=7, 10, and 15 mm (giving slab aspect ratios $4\leq A=R_{o}/d\leq 8.57$) were fabricated from polydimethylsiloxane (PDMS) (Sylgard 527; Farnell) with measured shear moduli in the range 770 ≤ *G* ≤ 1,720 Pa and Poisson’s ratio ν=0.5 (see *Materials and Methods* for more details). The elastomers were adhered to the inner boundaries of the rigid, open-topped molds in which they cured. The initial separation distance, or gap width b, between the substrate and the overlying glass plate was predetermined using spacers of a known size in the range 0.25≤b≤2.5 mm. The compliant Hele-Shaw cell was prefilled with a viscous fluid (glycerol; Sigma-Aldrich) whose viscosity was measured prior to each experiment and found to take values in the range 0.33≤μ≤1.20 Pa⋅s, depending on ambient-humidity conditions in the laboratory. The same fluid was injected into the prefilled cell through a port of 3 mm diameter in the center of the cell at prescribed volumetric fluxes in the range 10≤Q≤80 mL/min, using a syringe pump (Legato 111; KD Scientific).

Intruding fluid spread radially outward from the central source (displacing resident fluid toward the cell rim, which acted as a sink), was dyed for detection (forming a circle of radius $r_{f}$ in images taken from above) and imaged at 25 frames per second (fps) using a camera (Genie; Teledyne Dalsa). The axisymmetric substrate deformation from its initially flat, undeformed configuration was measured by monitoring the deflection of an initially straight line at the surface of the substrate that spanned a diameter of the cell. The line was imaged at 25 fps using a digital single-lens reflex camera (D7100; Nikon), positioned orthogonal to the line and inclined at 30° to the horizontal. Subpixel accuracy (to ±5 μm) was achieved by fitting a Gaussian profile to light-intensity values across the detected line (18).

To gain insight into the flow-induced deformation of the cell, we develop a simple scaling argument based on the fluid pressure inside the cell. In a large cell with a narrow gap, $R_{o}/b\gg 1$, the radial pressure gradient is proportional to the imposed flow rate through Darcy’s law, $Q=-\left[2\pi rb^{3}/\left(12\mu \right)\right]\;\partial_{r}p_{f}$, which provides a scale for the fluid pressure $p_{f}∼12\mu Q/\left(2\pi b^{3}\right)$. The radial pressure distribution in the fluid deforms the soft slab through shear. Assuming linear elasticity and homogeneous deformation on a length scale d, the pressure at the surface of the slab scales as $p∼Gw_{f}/d$, where G is the shear modulus and $w_{f}/d$ the material strain; $w_{f}$ is the vertical displacement of the surface of the elastic solid from its initially undeformed position, and d is the depth of the slab. Balancing fluid and elastic pressures, $p_{f}∼p$, we define a fluid–structure interaction (FSI) parameter, $F=12\mu Qd/\left(2\pi Gb^{4}\right)$, that provides a measure of the size of the deflection $w_{f}$ near the center of the cell compared with the initial gap thickness b.

In Fig. 1*B*, we compare deformation profiles for different values of the FSI parameter F as intruding fluid spreads through the cell (experimental measurements of the deformation are represented by dots; vertical, dashed lines indicate the radial position of the front of intruding fluid at different instants in time). For F<1 (Fig. 1*B*, *Top*), the deformation is small compared to the initial gap thickness and quickly reaches a constant profile, indicating a steady state. However, for F>1 (Fig. 1*B*, *Bottom*), the solid deformation in the vicinity of the injection port is comparable to the initial gap thickness, the deformation profile evolves as the fluid spreads, until a steady state is reached for t≃5 s, and bulging occurs near the cell rim.

To better understand the elastic bulging, we develop a mathematical model of this system. We model the axisymmetric flow of a viscous, incompressible fluid in the large, narrow cell ($R_{o}/b\gg 1$) with depth-averaged lubrication equations. Due to the flow-induced deformation of the slab, the gap width of the cell has a radial distribution $b+w_{f}\left(r,t\right)$, where $w_{f}\left(r,t\right)$ is the deflection of the slab surface. Volume conservation yields the following evolution equation for $w_{f}\left(r,t\right)$:

$${\dot{w}}_{f}=\frac{1}{12\mu }\nabla \cdot \left({\left(b+w_{f}\right)}^{3}\nabla p_{f}\right),$$ [1]

where $p_{f}\left(r,t\right)$ is the pressure in the fluid. Eq. **1** is subject to a source flux at the origin, $r{\left(b+w_{f}\right)}^{3}\partial_{r}p_{f}=-12\mu Q/2\pi$ at r=0, and an ambient-pressure sink at the cell rim, $p_{f}=0$ at $r=R_{o}$. Assuming that material strains are small, i.e., displacements u≪d, we use linear elasticity to model the axisymmetric deformation of the elastic slab:

$$\sigma =-pI+G\left(\nabla u+\nabla u^{T}\right),\;\nabla \cdot \sigma =0,\;\nabla \cdot u\;=\;0,$$ [2]

where σ denotes the stress, $u=ue_{r}+we_{z}$ is the displacement vector with radial and vertical components, p is the pressure, and I is the identity tensor. The elastic slab is subject to the no-slip condition on the inner surfaces of the rigid mold, u = 0 at z=d and at $r=R_{o}$. At the fluid–solid interface, z=0, $w=w_{f}$, $\sigma_{zz}=-p_{f}$, and $\sigma_{rz}=0$, whereas at the origin, r=0 and $u=\sigma_{rz}=0$.

Choosing the slab depth, d, as the characteristic length scale, a pressure scale based on the fluid pressure, $12\mu Q/\left(2\pi b^{3}\right)$, a scaling for displacement due to elastic stress, $12\mu Qd/\left(2\pi Gb^{3}\right)$, and an appropriate timescale, $12\mu d^{3}/\left(Gb^{3}\right)$, we nondimensionalize the governing equations (and denote dimensionless quantities with a hat). This yields

$${\dot{ŵ}}_{f}=\nabla \cdot \left({\left(1+F{ŵ}_{f}\right)}^{3}\nabla {\hat{p}}_{f}\right),$$ [3]

subject to $\hat{r}{\left(1+F{ŵ}_{f}\right)}^{3}\partial_{\hat{r}}{\hat{p}}_{f}=-1$ at $\hat{r}=0$ and ${\hat{p}}_{f}=0$ at $\hat{r}=A$, and

$$\hat{\sigma }=-\hat{p}I+\left(\nabla \hat{u}+\nabla {\hat{u}}^{T}\right),\;\nabla \cdot \hat{\sigma }=0,\;\nabla \cdot \hat{u}=0$$ [4]

subject to $\hat{u}=0$ at $\hat{z}=1$ and at $\hat{r}=A$, $ŵ={ŵ}_{f}$, ${\hat{\sigma }}_{\hat{z}\hat{z}}=-{\hat{p}}_{f}$ and ${\hat{\sigma }}_{\hat{r}\hat{z}}=0$ at $\hat{z}=0$, and $û={\hat{\sigma }}_{\hat{r}\hat{z}}=0$ at $\hat{r}=0$. Following nondimensionalization, only two free dimensionless parameters remain: the FSI parameter $F=12\mu Qd/\left(2\pi Gb^{4}\right)$ and the slab aspect ratio $A=R_{o}/d\gg 1$. We numerically solved this time-dependent system of coupled equations and also sought steady-state solutions in MATLAB (see *Materials and Methods* for more details).

**Fig. 2.**
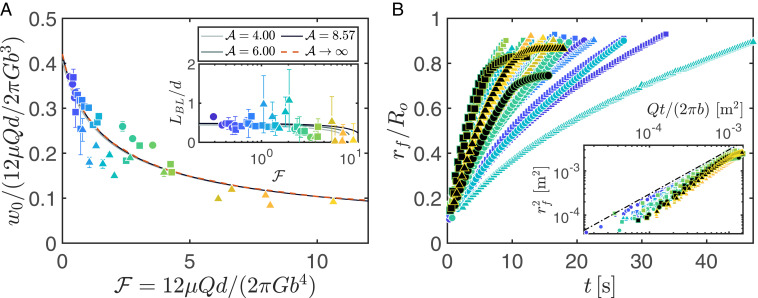
Deformation and flow in a soft cell. (*A*) The steady, flow-induced solid deformation at the center of the cell $ŵ=w_{0}/\left(12\mu Qd/2\pi Gb^{3}\right)$ as a function of the FSI parameter $F=12\mu Qd/\left(2\pi Gb^{4}\right)$. Experimental data are shown by markers, color-coded by the value of F; error bars indicate the standard deviation of measurements over a 2-s interval once steady state has been reached. Numerical predictions for different slab aspect ratios A are shown by the solid curves (as indicated in the legend); the curves are almost indistinguishable, however, indicating weak A dependence. The red, dashed line represents the solution for an effectively infinite cell, A→∞ with no lateral confinement. (*Inset*) The steady, lateral extent of the elastic boundary layer $L_{BL}=R_{o}-r_{bulge}$ near the cell rim where bulging occurs, scaled by the depth d of the soft solid, also as a function of F. (*B*) The radial position of the front of intruding fluid $r_{f}$ as a function of time t for various values of F, as indicated by the color of the markers (c.f. abscissa in *A*). The data markers for the experiments which resulted in choking are filled in black. (*Inset*) Raw data plotted in terms of the quantities that scale in a rigid Hele-Shaw cell, $r_{f}^{2}∼Qt/\left(2\pi b\right)$; this scaling is indicated by a black line. In both *A* and *B*, the shape of the data marker represents the aspect ratio of the elastic slab; A=4 (circles), 6 (squares), and 8.57 (triangles). Experimental and numerical data are available in the Dataset S1.

Good agreement is found between the predicted deformation profiles (solid lines) and experimental measurements (dots), as shown in Fig. 1*B*. Fig. 2*A* shows the dimensionless flow-induced deformation at the cell center ${ŵ}_{0}$ as a function of F, once a steady state has been reached (i.e., for $\hat{t}=t/\left[12\mu d^{3}/\left({Gb}^{3}\right)\right]\gg 1$). The results confirm that greater deformation occurs for increasing flow rate or cell compliance and reveals a very weak dependence of the central deflection on the slab aspect ratio A. Indeed, there is good agreement between all of the experiments and the solution for an effectively infinite cell without lateral confinement (shown by the red, dashed line), which indicates that the central deformation of the slab is predominantly resisted by local shear forces due to the adhesion to the bottom of the rigid mold, rather than by the lateral confinement at $r=R_{o}$.

Our results indicate that bulging near the outflow is a consequence of 1) the radial pressure profile in the fluid, which deforms the elastomer through shear; 2) the incompressibility of the soft solid; and 3) the spatial constraints imposed by the rigid enclosure. As the elastomer is adhered to the base of the confining mold, a parabolic displacement profile (analogous to Poiseuille flow) is established in the solid and displaces material toward the rim of the cell (indicated schematically in Fig. 1*A*). Bulging occurs within an elastic boundary layer where material experiences the lateral confinement of the inner wall of the rigid mold, at $r=R_{o}$. A metric for the boundary layer is given by the distance of the bulge peak from the cell rim, $L_{BL}=R_{o}-r_{bulge}$, where $-w_{f}\left(r_{bulge}\right)$ is the steady-state bulge height, and consequently, $b_{min}=b+w_{f}\left(r_{bulge}\right)$ is the minimum gap. The size of the boundary layer is comparable to the initial depth of the slab $L_{BL}/d=O\left(1\right)$ (Fig. 2*A*, *Inset*) but does exhibit some dependence on A.

Having discussed the solid deformation of the cell, we now turn our attention to spreading of fluid through the cell, which drives the evolution of the bulge to a time-independent profile, by examining the radial position of the front of intruding fluid $r_{f}$ as a function of time (Fig. 2*B*). Recalling that in the case of a rigid cell, conservation of volume indicates that $r_{f}^{2}b∼Qt$, we scale the raw data accordingly in Fig. 2*B*, *Inset*. For small values of F, the slab is relatively undeformed, and the front propagation is comparable to that found in a rigid cell. Large flow-induced deformation alters the rate of front propagation from that experienced in a rigid cell because the fluid must first fill the volume left vacant by displaced elastomer before spreading out into a narrowing gap.

In Fig. 2*B*, the experiments indicated by filled black markers exhibit a sudden departure from the early-time scaling, whereby $r_{f}$ tends to a constant value. This is indicative of choking of the flow—a phenomenon previously observed in collapsible elastic tubes (19)—which is a consequence of the bulge near the cell rim making contact with the cell roof, blocking the outflow and preventing fluid from escaping the cell. In practice, when choking occurs, the pressure inside the cell diverges until the syringe pump driving the flow stalls. Solid material then remains adhered to the cell roof until a reset is performed by manually detaching the elastomer. (In the numerics, choking similarly manifests as the minimum gap shrinking to zero at finite time in the time-evolution simulations and as the lack of existence of a steady-state solution).

**Fig. 3.**
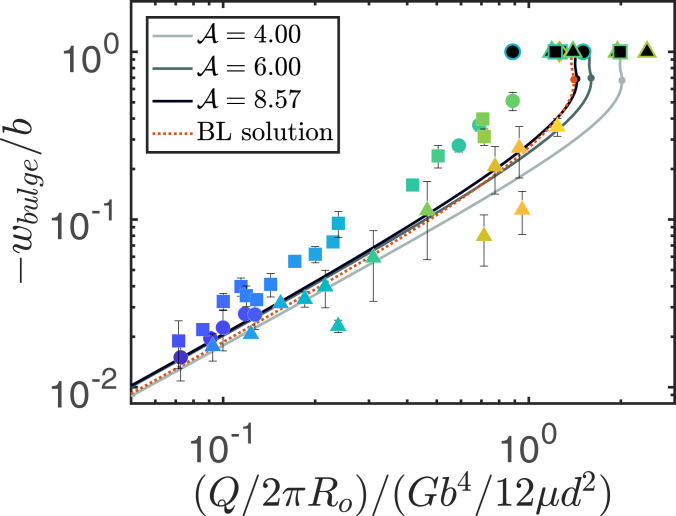
Choking a compliant cell. The steady, dimensionless bulge height $-w_{bulge}/b=1-b_{min}/b$, where $b_{min}$ is the minimum gap, as a function of the imposed injection flux, nondimensionalized using the critical scale, $Q/Q_{c}*=\left(Q/2\pi R_{o}\right)/\left(Gb^{4}/12\mu d^{2}\right)=F/A$. Experimental results are shown with colored markers: the shape of the data marker represents the aspect ratio of the elastic slab; *A* = 4 (circles), 6 (squares), and 8.57 (triangles), and the color indicates the value of F following the scheme in Fig. 2*A*. Sufficiently large $Q/Q_{c}*$ results in cell closure (indicated by black-filled markers); the bulge makes contact with the cell roof, $-w_{bulge}/b=1$, which chokes the flow completely, interrupting the experiment. The lines indicate numerical steady-state solutions for each of the experimental aspect ratios. Each steady-solution branch turns back on itself at the dot on the line (maximal F), so that no steady solution exists for values of $Q/Q_{c}*$ beyond this threshold. The red, dotted line represents the boundary-layer (BL) solution for an infinitely long, 2D slab bounding a channel with an imposed flux $Q_{2D}=Q/2\pi R_{o}$ (per unit length). As the bulge height increases, the steady state becomes linearly unstable at the turning point. Beyond this value (while $-w_{bulge}/b<1$, since no steady solution can exist with zero gap), the steady state restabilizes at least once over a small height range. However, even in this region, the steady state was found to be sensitive to a small 1% perturbation, which explains why none of the steady states with a bulge height ≳0.7b (and a flux up to 3% less than the maximum value) were observed in the experiments or time-evolution simulations. Experimental and numerical data are available in the Dataset S1.

We examine the threshold for choking in Fig. 3 and develop a simple scaling argument based on material strains in the elastic boundary layer near the cell rim. In the case of a constant volumetric injection flux, the total outflux at the rim is equal to the imposed injection flux Q. The elastic boundary layer is contained within an axisymmetric ring near the cell rim of cross-section $x=R_{o}-r∼d$ and z∼d through which fluid flows with flow rate $Q/2\pi R_{o}$ per unit length. The rim flux across this region is driven by horizontal pressure gradients, $\partial_{r}p_{f}∼p_{f}/d$, such that $Q/\left(2\pi R_{o}\right)∼b^{3}p_{f}/\left(12\mu d\right)$. For the cell to completely choke, the vertical solid displacement must be equal and opposite to the initial gap thickness, so that $b+w_{f}=0$. The pressure required to achieve such a material strain scales as p∼Gb/d, and, by balancing $p_{f}∼p$, we find that the critical volumetric flux required to choke a compliant Hele-Shaw cell is an O(1) multiple of

$$Q_{c}^{*}=2\pi Gb^{4}R_{o}/\left(12\mu d^{2}\right).$$ [5]

The results in Fig. 3 show that the steady-state bulge height is linearly proportional to $Q/Q_{c}^{*}=F/A$, for $Q/Q_{c}^{*}≲1$. The gap collapses to zero, choking the flow, for $Q/Q_{c}^{*}≳1$. Since the bulging responsible for flow choking occurs within an O(d) distance to the rim, it forms part of a boundary-layer solution that does not depend on the injection region and the radial geometry. The equations for the boundary-layer solution describe the deformation of an infinitely long, two-dimensional (2D) slab bounding a channel with an imposed flux $Q_{2D}=Q/2\pi R_{o}$ (per unit length), and numerical solution of those equations shows that the cell completely chokes when the local flux exceeds $Q_{2D}\approx 1.4Gb^{4}/12\mu d^{2}$, which agrees well with the experiments and numerics in the radial system (see red, dotted curve in Fig. 3). The boundary-layer solution implies that choking will occur at this critical local flux regardless of the overall geometry of the cell. We note that, in experiments with an off-center injection into a circular cell, contact was observed to first occur ahead of the front region closest to the cell rim, before spreading around the circumference of the cell and choking the flow everywhere. However, in a rectilinear channel, for example, the influence of bounding side walls may inhibit solid deformation and impede complete closure.

When the system chokes, full contact is achieved between the bulge and the cell roof. This is in contrast to previous studies of soft, lubricated contact (20–22), which demonstrated that repulsive lubrication forces can induce elastic deformation and thus prevent full contact being reached. Here, for prescribed fluxes above the critical value, $Q/Q_{c}^{*}≳1$, the mechanism for flow choking is robust because it involves a positive-feedback loop. As the gap reduces, fluid squeezing through the narrow constriction causes an increase in the local pressure gradient (as in the case in bearings) (23, 24). This pressure gradient pushes the solid toward the rim, but the lateral confinement redirects the pressure force upwards, thus reducing the gap further. As the gap shrinks toward zero, the pressure squeezing the channel closed diverges toward infinity. (An equivalent explanation is that, due to the incompressibility of both solid and fluid, the rim acts as a constant-flux sink that sucks the fluid out, but when the gap shrinks and the fluid flux cannot keep up, solid material is instead sucked up and chokes the cell.)

Following contact between the bulging elastomer and the cell roof, if the pressure continues to rise (without the syringe pump stalling), then the bulge can be forced out into the channel beyond the bounding enclosure, forming an extruded seal. Our fluidic fuse fails if a leaking path forms in this seal, which can occur as the result of elastic deformation (25) or if localized fracture occurs and a crack forms that permits the escape of fluid. We therefore recommend that toughened elastomers (26) or hydrogels (27), which exhibit large-strain reversible deformation, are used in applications to alleviate the possibility of fracture and subsequent leakage.

We note that although a system driven by a fixed pressure drop instead of a fixed flux has the same steady-state solutions, its dynamics are different. Since a divergent pressure is required to achieve contact between the bulge and cell roof, a pressure-driven system will, in theory, not choke. Instead, numerical simulations show that the system approaches a unique steady state for each value of the pressure drop, with the bulge height $-w_{bulge}$ increasing monotonically toward b as the pressure drop diverges. In practice, however, for sufficiently large driving pressure, the remaining gap $b_{min}=b+w_{bulge}$ collapses due to small-scale adhesive forces and/or molecular effects, and the system chokes. The competition between adhesive forces and lubrication pressure, which may result in choking of pressure-driven systems, is beyond the scope of this study.

In conclusion, a compliant Hele-Shaw cell, comprising a rigid plate and an enclosed, soft solid separated by a narrow gap, exhibits behavior that is analogous to that of an electrical fuse. The threshold for choking the flow depends entirely on the physical parameters of the system; this mechanical method of interrupting the flow could therefore be engineered into microfluidic devices as a passive flow limiter. Provided the soft solid does not fracture, the elastic system can be manually reset to the initially undeformed position (i.e., detached from the cell roof) and used again, much like a circuit breaker. Here, we demonstrated fuse-like behavior in a fluidic circuit using a fluid (glycerol) of comparable viscosity to the viscous liquids used in flow-focusing devices (28) and, in particular, to the prepolymer solutions used in the microfluidic production of polymeric droplets (29).

**Fig. 4.**
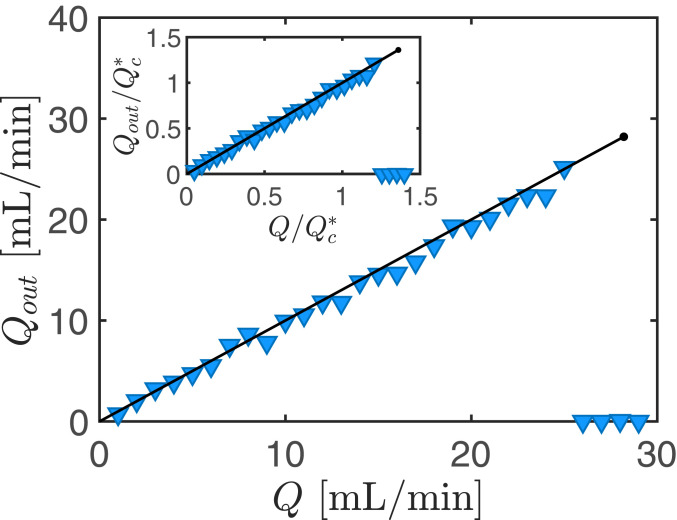
A fuse-like response with low-viscosity fluids. The measured outflux $Q_{out}$ as a function of the imposed volume flux Q of water (μ=1 mPa⋅s) injected into a small-scale Hele-Shaw cell; $R_{o}=30$ mm, d=2 mm, b=100 μ m, and G=880 Pa. Experimental and numerical results are shown with markers and a solid line, respectively. In the experiments, when the imposed injection flux is too large, the cell chokes and the outflux (as well as the actual injection flux) drops to zero. In the numerics, beyond a critical value (indicated by the dot) no steady-state solutions exist. (*Inset*) The raw data presented in dimensionless form following rescaling by $Q_{c}*=2\pi Gb^{4}R_{o}/\left(12\mu d^{2}\right)$. Experimental and numerical data are available in the Dataset S1.

However, many microfluidic applications use fluids with much lower viscosity than that of glycerol, which would result in a much larger critical flux for choking (Eq. **5**). Keeping the critical flux low is best achieved through a reduction in channel thickness (as it enters Eq. **5** with a fourth power), with the proviso that the gap remains sufficiently large to prohibit spontaneous adhesive contact (as occurs in lithographic stamp collapse) (30). To demonstrate that flow-induced choking can be realized with low-viscosity fluids, we developed a small-scale version of our experiment with $R_{o}=30$ mm, d=2 mm, b=100 μm, and G=880 Pa. Using water as the working fluid (μ=1 mPa⋅s), we measured the outflux $Q_{out}$ as a function of the imposed injection flux Q (Fig. 4). The data show a clear choking threshold, which agrees well with our numerical results and the theoretical scaling (Eq. **5**). Below the threshold, the cell permits the imposed flux to pass through ($Q_{out}=Q$), but if the imposed flux is above the threshold, then the outflux (as well as the actual injection flux) drops to zero. Further investigation into the effects of boundaries in rectilinear geometries is required if this fuse-like behavior is to be exploited in conventional microfluidic geometries.

At the other end of the length-scale spectrum, the results presented here may provide valuable insight into industrial flows present in oil recovery and carbon sequestration. The Hele-Shaw cell is the archetypal experiment for recreating, and visualizing, flow conditions found in oil fields in the laboratory (31), and this study provides a platform on which to build further knowledge of the poroelastic response of rock formations to pressurized fluid injections. The Hele-Shaw cell is also the cornerstone of research into viscous-fingering instabilities in two-phase flows (32, 33). Recently, attention has turned to controlling, or suppressing, the interfacial instability, which occurs when a less-viscous fluid intrudes into a more-viscous fluid. Typically, flow control is achieved via modifications of the geometry of the cell, for example, tapering the cell geometry in the flow direction (34–36), increasing the plate separation as a function of time (37, 38), or replacing the top plate with an elastic membrane that can inflate (39–43). Our findings indicate that compliant, confined boundaries provide further opportunities for flow control.

## Materials and Methods

### Experiments.

The soft slabs were fabricated from PDMS (Sylgard 527; Farnell). Specifically, the base polymer and catalyst were mixed in a 1:1 ratio, degassed in a vacuum chamber, and then left to cure for 1 wk at room temperature inside rigid, circular molds (made from plastic) that were glued to a glass base plate. The shear moduli of the resultant elastomers were measured using oscillatory shear tests in a rheometer (Kinexsus Pro+; Malvern Panalytic) and found to be in the range 770 ≤ *G* ≤ 1,720 Pa. The axisymmetric substrate deformation was measured by imaging a line spanning the surface of the slab through the base of the mold. The line consisted of fluorescent tracer particles (Fluostar; EBM Corporation) embedded in the surface of the slab and was illuminated using a green-light laser. The particles had a rhodamine B coating that fluoresced red, and a long-pass optical filter was positioned in front of the camera to reduce background noise in the detected images. The radius of the injection port was 1.5 mm, which is small compared to the horizontal length scale of the central boundary layer, and therefore had negligible influence on the reported FSI. To measure the outflux as a function of the input volume flux, the small-scale Hele-Shaw device was positioned on top of a precision balance (FKB-8; Kern), which recorded the mass of fluid that exited the device over a 10-s interval.

### Numerics.

A stream-function formulation $û=-\left(1/\hat{r}\right)\partial_{\hat{z}}\hat{\Psi }$, $ŵ=\left(1/\hat{r}\right)\partial_{\hat{r}}\hat{\Psi }$ was employed to enforce the incompressibility in the elastic Eq. **4**. The resulting equation and boundary conditions for Ψ were discretized in MATLAB using a finite-difference method on a square grid with spacing 0.02 in the domain $0\leq \hat{z}\leq 1$, $0\leq \hat{r}\leq A$ to yield a linear coupling between ${ŵ}_{f}$ and $\partial_{\hat{r}}{\hat{p}}_{f}$. A fully implicit forward Euler method was then used to evolve the system forward in time using the lubrication Eq. **3**, with a slowly increasing time step. The resulting late-time steady states agreed with steady-state solutions obtained by Newton iteration. The results changed by less than 2% when the spatial or temporal resolution was doubled; the deformation away from the center or rim was verified to agree well with the prediction $u\approx \left(z^{2}-d^{2}\right)\partial_{r}p_{f}/\left(2G\right)$ from an asymptotic analysis of the solid analogous to fluid lubrication theory, and the initial deformation was verified to grow and spread like $t^{1/3}$ from the injection point, as predicted by seeking a self-similar solution of the equations. The parabolic displacement profile was also used as far-field boundary condition at r=10d for an “infinite” cell (Fig. 2*A*) and at $R_{o}-r=10d$ for the rim boundary-layer solution (Fig. 3) with a 2D geometry. For the states with bulge height ≳0.7b, a stretched grid with spacing down to 0.001 was used to properly resolve the bulge near the rim, and the steady-state solutions were calculated using the pressure drop as the control parameter instead of the flux for stability reasons. Once found, these states were perturbed by reducing the deformation by 1% and then evolved forward in time with a fixed flux, and the subsequent growth of the perturbation was taken as confirmation that the states are sensitive to perturbations.

## Supplementary Material

Supplementary File

## Data Availability

All study data are included in the article and supporting information.
